# Anthracycline‐induced cardiotoxicity on regional myocardial work and left ventricular mechanical dispersion in adolescents and young adults in post‐lymphoma remission

**DOI:** 10.1002/cam4.6857

**Published:** 2024-01-10

**Authors:** Mohamed Jaber, Alexandre Armand, Emmanuelle Rochette, Severine Monzy, Victoria Greze, Justyna Kanold, Etienne Merlin, Justine Paysal, Stéphane Nottin

**Affiliations:** ^1^ CHU Clermont‐Ferrand, Pédiatrie Générale Clermont‐Ferrand France; ^2^ CHU de Clermont‐Ferrand, Service Hématologie Oncologie Pédiatrique Clermont‐Ferrand France; ^3^ Université Clermont Auvergne, INSERM CIC 1405, CRECHE Unit Clermont‐Ferrand France; ^4^ Cardiologue libéral, Pôle Santé République Clermont‐Ferrand France; ^5^ CHU Clermont‐Ferrand, Néonatologie et Réanimation Pédiatrique Clermont‐Ferrand France; ^6^ Laboratory of Cardiovascular Adaptations to Exercise Avignon France

**Keywords:** anthracycline, childhood lymphoma survivors, intraventricular mechanical dispersion, myocardial work, regional myocardial function, speckle tracking echocardiography

## Abstract

**Background:**

Myocardial work (MW) is a new echocardiographic tool with a high sensitivity to detect early and subtle alterations of myocardial function. We aimed to evaluate the late effects of anthracyclines by assessing the global and segmental MW and intraventricular mechanical dispersion from speckle tracking echocardiography in childhood lymphoma survivors (CLS).

**Methods:**

Thirty‐one young adults including CLS and age‐matched healthy controls were enrolled. All underwent echocardiography including an evaluation of left ventricular (LV) morphology and regional function. We assessed LV longitudinal (differentiating sub‐endocardial and sub‐epicardial layers), circumferential strains and twist, global and regional MW index (MWI). LV mechanical dispersion was assessed from the time dispersion of LV longitudinal strain, from myocardial wasted work (MWW) and myocardial work efficiency (MWE).

**Results:**

The longitudinal strains both at the level of the sub‐endocardium and sub‐epicardium were reduced in CLS compared to controls. The global MWI was also decreased (1668 ± 266 vs 1870 ± 264%.mmHg in CLS patients and controls, respectively, *p* < 0.05), especially on the apical segments. An increase of LV intraventricular mechanical dispersion was observed in CLS. MWW and MWE remained unchanged compared to controls.

**Conclusion:**

Our results strongly support that cardiac remodeling is observed in CLS, characterized by a decrease in MW and an increase in LV mechanical dispersion. The apex is specifically altered, but its clinical significance remains uncertain. MW as a complement to strain seems interesting in cancer survivors to detect myocardial dysfunction at early stage and adapt their follow‐up.

## Graphical abstract

Cardiac remodeling is observed in childhood lymphoma survivors, characterized by a modification in myocardial work and left ventricular mechanical dispersion. Myocardial work seems interesting in cancer survivors to detect myocardial dysfunction at an early stage and thus to adapt their follow‐up and management.

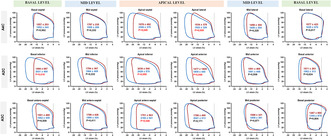

## 1 INTRODUCTION

Childhood cancer is a heterogeneous group. Among adolescents, the most common cancer group is lymphoma (27%). In most European and North American countries, survival exceeds 80% at 5 years,^1^, ^2^ especially with chemotherapy, which usually combines antineoplastic drugs whose anthracyclines. Current international protocols aim to reduce the intensity of chemotherapy without threatening survival, in order to limit its toxicity, particularly on the cardiovascular system. Indeed, cardiac adverse effects are one of the main causes of mortality among childhood cancer survivors, especially with anthracyclines which are associated with an increased risk for heart failure compared to their siblings, with an increasing incidence of prolonged survival.^3^, ^4^, ^5^ The appearance of chemotherapeutic cardiotoxicity may indicate the interruption of treatment,^6^, ^7^ and therefore cardiotoxicity is an important prognostic factor.

Cardiology and oncology societies presented clinical guidelines on the prevention and treatment of anthracycline‐induced cardiotoxicity (AIC) focused on screening for left ventricular ejection fraction (LVEF) reduction throughout the treatment.^8^, ^9^ Recently, the American Society of Echocardiography and European societies recommended the Global Longitudinal Strain (GLS), from speckle tracking echocardiography (STE), as a marker of myocardial dysfunction.^8^, ^9^, ^10^, ^11^ Indeed, it may be associated with an early sign of evolving cardiomyopathy among childhood cancer survivors treated with anthracyclines despite their normal LVEF.^12^, ^13^, ^14^, ^15^ However, GLS represents only one component of LV strains. A comprehensive approach to assess LV systolic function needs to combine GLS with circumferential strains,^16^ and also LV twisting mechanics.^17^ During systole, due to the orientation of the myocardial fibers, the base and the apex rotate in opposing directions, inducing LV twists, maintaining an essential part of systolic function and could be reduced in childhood lymphoma survivors (CLS).^17^, ^18^, ^19^ Another important feature is to propose a regional assessment of LV strain^16^ and differentiate subendocardial and subepicardial layers that could provide additional information on the subtle myocardial remodeling in CLS.

Importantly, despite its capability to assess LV regional myocardial function, evaluation of LV strains remains limited by its dependence on loading conditions of the heart.^20^ To overcome this limitation, a new echocardiographic tool based on the evaluation of myocardial work (MW) has been developed to detect subtle LV dysfunctions.^21^, ^22^, ^23^ Initiated by the works of Russel et al., MW is obtained by integrating both cardiac deformation and noninvasive intra‐ventricular pressure.^24^, ^25^ In adult populations, MW has already been used in many diseases and previous work demonstrated its better sensitivity in predicting coronary artery disease and assessing early subclinical ischemic myocardial dysfunction compared to GLS.^26^ Notably, MW can be assessed regionally, according to an 18‐segments model of the LV. However, both its global and regional assessment has never been explored in CLS and remains to be evaluated.

Myocardial performance can also be altered by an uncoordinated shortening of LV myocardial fibers, which could lead to LV dyssynchrony (LVD) characterized by an increased mechanical timing dispersion at end‐systole.^19^ In childhood cancer survivors, structural myocardial abnormalities with the presence of diffuse interstitial fibrosis, and sarcomeric disruption are evidenced.^5^, ^27^ Myocardial fibrosis is usually associated with conduction disorders, so we questioned a potential increase of LV mechanical dispersion in CLS.^28^ Segmental analysis of the timing of LV strains is one way to assess LV mechanical dispersion, and MW is another. Indeed, a study of MW, which includes myocardial constructive (MCW) and wasted work (MWW), could reveal an increase in mechanical dispersion via an increase in MWW.^19^, ^29^

In this context, the objective of the study was to compare the cardiac function of CLS, with healthy age‐matched controls, to determine the late effects of anthracyclines by assessing not only myocardial strain, but also MW and LV mechanical dispersion. We hypothesized that CLS treated with anthracyclines have adverse cardiac remodeling characterized by (1) a decrease of longitudinal strain, especially in the subendocardial layer, (2) a decrease of segmental and global MW, (3) increase of LV intraventricular mechanical dispersion associated with an increase of MWW.

## 2 METHODS

### 2.1 Study design and population

This study collected the data of our previous prospective cross sectional case–control study run from January 2022 to May 2022 in Clermont‐Ferrand University Hospital, France.^30^ Sixteen CLS were included and compared with 15 healthy controls, recruited from the database of the Centre d'Investigation Clinique of Clermont‐Ferrand University Hospital, and matched for sex, age (± 5 years), and body mass index (BMI ± 5 kg/m^2^). None of the participants had cardiac medication at the time of evaluation and all had electrocardiogram (ECG) recording and were in sinus rhythm. CLS aged between 18 and 25 years old were followed for childhood lymphoma post‐therapeutic surveillance, and considered as recovered (i.e., free of any lymphoma medication or radiotherapy for at least 1 year), with no physical disability at the day of inclusion. To guarantee a late cardiac functional assessment and the absence of any progressive malignancy, only patients who have recovered from treatment for over 1 year were eligible for the study. Subjects were excluded if they had been clinically diagnosed with a running infection or undergone corticosteroid treatment for at least 1 week within the last month before the day of visit.^30^, ^31^

### 2.2 Anthropometrics

Height was determined using a mural stadiometer with the subject barefoot (graded in cm, precision of 0.5 cm), and weight using a digital weight scale (graduated in kg, with 0.1 kg precision), permitting calculation of BMI (body weight/body height^2^, with weight in kg and height in m). Blood pressure (BP) were recorded at rest, using an automatic device (General Electric Dynamap PRO 300 V2, Boston, MA, USA).

### 2.3 Biological data

Blood samples were collected after 12 h of fasting and at least 10 min resting. The two main cardiotoxicity biomarkers, troponin I and N‐terminal pro‐brain natriuretic peptide (NT‐proBNP), were assessed in all participants.^3^ Standard blood test as hemoglobin, C‐reactive protein, thyroid hormones and albumin were also performed to identify irregularities, which could influence cardiovascular parameters.^16^

### 2.4 Echographic conventional recordings

Echocardiography was carried out with the subject in the left lateral decubitus position, with Vivid ultrasound systems (GE Healthcare, Horten, Norway) using a 3.5 MHz transducer (M4S probe), by the same, experimented, cardiologist, blinded of the status of patients. 2D echocardiographic measurements were made in accordance with the American Society of Echocardiography guidelines.^32^ Cine loops are stored in one R–R interval length to facilitate analysis. An ECG signal is recorded for R–R interval clip storage and no ambiguous ECG complexes or leading T‐waves were displayed. Thickness parameters were measured from the parasternal long axis view. LV volumes and LVEF were assessed using the Simpsons biplane method. Stroke volume and cardiac output were assessed from a 5‐chamber view and then indexed to body surface area. LV mass was estimated by the Lang formula and indexed to body surface area. Diastolic function was assessed by Doppler from peak early (E) and atrial (A) trans‐mitral flow velocities. Septal and lateral E' wave from tissue Doppler completes this evaluation. The LV filling pressures were evaluated from the E/E' ratio.

### 2.5 Two‐dimensional strain analysis from STE

Cine loops were recorded in parasternal long and short axis (basal and apical) and apical (5, 4, 2, and 3 chambers) views and saved for blinded off‐line analysis (EchoPAC, BT204 Version, GE Healthcare), with ECG recording. Time was expressed as a percentage of systolic duration avoiding therefore bias from differences in heart rate and cine loop frame rates. We used apical 4‐, 2‐, and 3‐chambers views to assess LV regional strains and time to peak (TTP) strains to obtain an18‐segments model, in accordance with the nomenclature of Lang et al.^32^ A segment‐by‐segment validation must be done to continue the analysis. When analysis was not possible or unreliable, the segment or loop were excluded from the analysis. Time‐derivation of longitudinal strains results in both systolic and diastolic strain rates. Notably, the diastolic GLS‐rate was used as an indicator of LV relaxation.^33^

LV twisting mechanics were assessed by STE using images from the apical and basal short‐axis views. Basal planes were assessed regarding the mitral valve and apical planes toward the apex with no visible papillary muscles. With LV contraction, the cardiac apex and the base rotates in counterclockwise and clockwise directions, respectively, when viewed from the apex. Twist represents the mathematical difference in the rotation of the apex and base, at each percentage of systolic duration.^13^ Peak systolic twisting velocity was defined by the peak positive rate of torsional deformation, whereas the peak diastolic untwisting velocity was defined by the peak negative rate of torsional deformation.^34^ Untwisting velocity is thought to be an important initial manifestation of active relaxation.

The LV mechanical dispersion was quantified by calculating the standard deviation (SD) of TTP systolic strain in the longitudinal direction in the 18‐segments model (SD_18S_), and the maximum delay between the segments with the earliest and the latest TTP (Delta_1‐18_).^19^

### 2.6 MW

MW was estimated using the Automatic Function Imaging (AFI) of the EchoPAC software (Version 203; GE Healthcare). MW was assessed by the combination of LV strain data (recorded on the apical 4‐, 3‐, and 2‐chambers views) obtained by STE, as a function of time throughout the cardiac cycle, and a noninvasively estimated LV pressure curve as previously described.^24^ The valvular markers of mitral and aortic opening and closure were taken first on the Doppler from peak early (E) and atrial (A) trans‐mitral flow and trans‐aortic, for reproducibility, and then imported into the MW analysis with a secondary modification on the 2D section for synchronization. All data are averaged on 2 cardiac cycles, synchronized with ECG, and we assumed that the high and low pressure measured was respectively equivalent to peak systolic and diastolic LV pressures and are uniform throughout the ventricle. Strain and MW evaluation was done by a single investigator. The global myocardial work efficiency (MWE) depends on the MCW and MWW, and was obtained as follows:

$$\mathrm{MWE}=\frac{\mathrm{MCW}}{\mathrm{MCW}+\mathrm{MWW}}\times 100$$

In systole, the “positive” work performed by the myocardium during shortening represents MCW, including lengthening in isovolumic relaxation, whereas the “negative” work performed by the myocardium during lengthening, including shortening in isovolumic relaxation, represents the energy loss, which is defined as MWW.^19^ For both global and segmental analyses, we constructed LV pressure‐strains loops. The area of the loops represented the Myocardial work index (MWI).

### 2.7 Statistical analysis

All values were expressed as mean ± standard deviation. Statistical analyses were performed using Medcalc (version 19.1, Medcalc Software). One‐way analysis of variance (ANOVA) was used to compare groups, after checking the normality of distribution of each variable by the Shapiro–Wilk test. In the absence of normal distribution, the nonparametric Kruskal‐Wallis test was used. Statistical significance for all analyses was assumed at *p* < 0.05.

## 3 RESULTS

### 3.1 Participants characteristics

**FIGURE 1:**
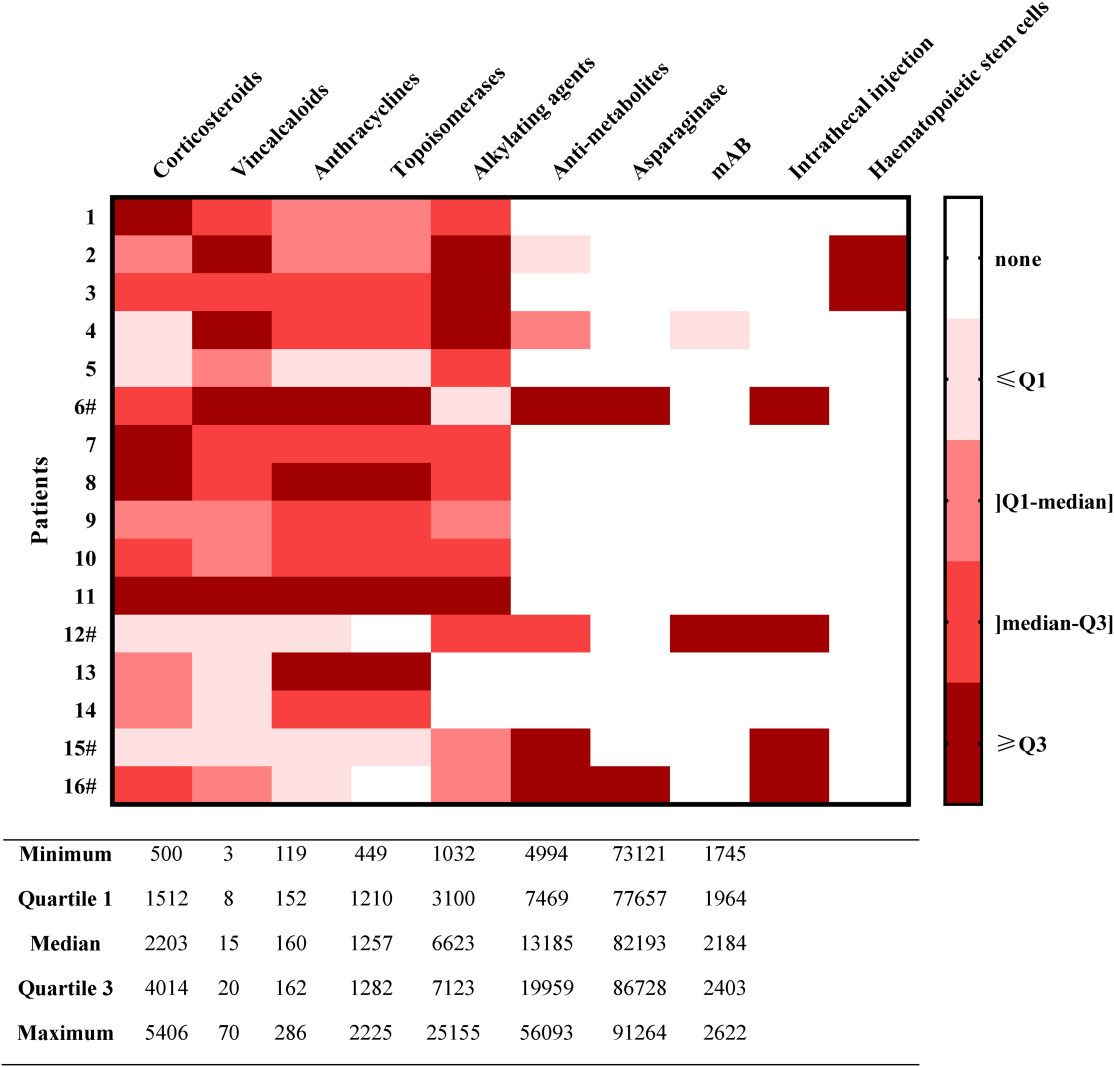
Heatmap for treatment exposure. Cumulative doses in mg/m^2^. Corticosteroids (prednisone or dexamethasone) are recorded as prednisone‐equivalent. Vinkaalcaloids: vinblastin, vincristin, vinorelbin. Anthracyclines: daunorubicine or doxorubicine. Type II topoisomerase inhibitor: etoposide. Alkylating agents: bendamustin, carmustin, cyclophosphamide, dacarbazin, ifosfamide, melphalan or procarbazin. Anti‐metabolites: cytarabin, gemcitabin, 6‐mercaptopurin, methotrexate or 6‐thioguanin. Monoclonal antibodies (mAb): brentuximab or rituximab. # Non Hodgkin lymphomas.

Clinical data of the two populations are presented in Table 1. The mean time interval between the end of chemotherapy and patient enrolment was 6.3 years. CLS were treated by chemotherapy with a mean cumulative dose of anthracyclines of 160 mg/m^2^ with a maximal dose of 286 mg/m^2^ in one patient. Doxorubicin was mainly used. Mean cumulative corticosteroids dose was 2600 mg/m^2^. Alkylating‐agent and type II topo‐isomerase inhibitors were used for all patients. Four Hodgkin lymphoma survivors had a total of 19.6 Gy mediastinal and cervical targeted radiation therapy (depending on diagnosis stage or relapse). In the course of therapy, we noted only two relapses, respectively 15 and 24 months after the date of diagnosis. These two were Hodgkin disease and with the same extension (stade 2A) with no spreadings, and beneficiated of autologous hematopoietic stem cell transplantation. A heatmap for individual treatment exposure are presented in Figure 1.

**TABLE 1:** Baseline Characteristics of Patients and Controls.

	Patients (*n* = 16)	Controls (*n* = 15)
Age	21 ± 2	23 ± 1,5*
Male (*n*)	9	7
Female (*n*)	7	8
BMI	23,5 ± 3,2	22,3 ± 2,5
Heart rate at rest (bpm)	81 ± 13	70 ± 13*
Systolic BP (mmHg)	118 ± 12	119 ± 16
Diastolic BP (mmHg)	76,6 ± 9,6	74,9 ± 8,8
Mean BP (mmHg)	89,5 ± 10,3	88,0 ± 7,8
Hodgkin disease	12	
Non‐Hodgkin lymphomas #	4	
Age at diagnosis (years)	14,7 ± 1,5	
Cumulative dose of Anthracyclines (mg/m^2^)	160,3 ± 37,4	
Time since end of treatment (years)	6,3 ± 2,4	
Radiotherapy (*n*)	4	
Hematopoietic stem cells transplantation for relapse (*n*)	2	
Other chemotherapy	All	

*Note*: Quantitative variables are expressed as mean ± standard deviation. *: significantly different from Controls (*: *p* < 0.05). # Non Hodgkin lymphomas gather, one B and one T lymphoblastic lymphoma, one Gray zone lymphoma and one large B cells lymphoma.

Abbreviation: BMI, body mass index.

There was no significant difference between CLS and healthy controls in term of BP. No patient had symptoms of heart failure. Heart rate was significantly higher in CLS compared to controls. No statistical difference was shown on troponin I and NT‐proBNP. A mean NT‐proBNP value of 51 ± 33 ng/L was found in CLS. Hemoglobin was higher in CLS. None had anemia. Nonsignificative difference was found on CRP, normal for all, thyroid hormone nor on nutritional status estimated by albumin levels.

### 3.2 Left ventricular morphology and function

Data of LV morphology and function are presented in Table 2. LV posterior wall thickness, LV septum thickness, LV end‐diastolic and end‐systolic volume, LV mass and LV mass indexed to body surface area were comparable in both populations. LVEF was significantly decreased in CLS but no LVEF below 60% was detected. There was no significant difference in the early to late diastolic filling ratio (E/A ratio) and E/E' ratio.

**TABLE 2:** Left ventricular morphology and function.

	Patients (*n* = 16)	Controls (*n* = 15)
LV Morphology		
LV septum thickness (cm)	0.84 ± 0.12	0.85 ± 0.17
LV posterior wall thickness (cm)	0.85 ± 0.16	0.91 ± 0.11
LV end‐diastolic volume (mL)	114 ± 32	106 ± 27
LV end‐systolic volume (mL)	33 ± 9	32 ± 8
LV mass (g)	145 ± 48	143 ± 38
LV mass/BSA	80 ± 11.5	83 ± 9.7
LV systolic function		
Ejection fraction (%)	65.2 ± 5.3 *	69.6 ± 6.3
LV diastolic function		
E wave (m/s)	0.86 ± 0.14	0.89 ± 0.15
A wave (m/s)	0.51 ± 0.08	0.57 ± 0.13
E/A	1.72 ± 0.33	1.59 ± 0.29
E/E' lat	4.5 ± 0.7	4.5 ± 0.9

*Note*: Values are mean ± standard deviation. *: significantly different from Controls (*: *p* < 0.05).

Abbreviations: BSA, body surface area; LV, left ventricular.

### 3.3 Left ventricular regional strains and MW

**FIGURE 2:**
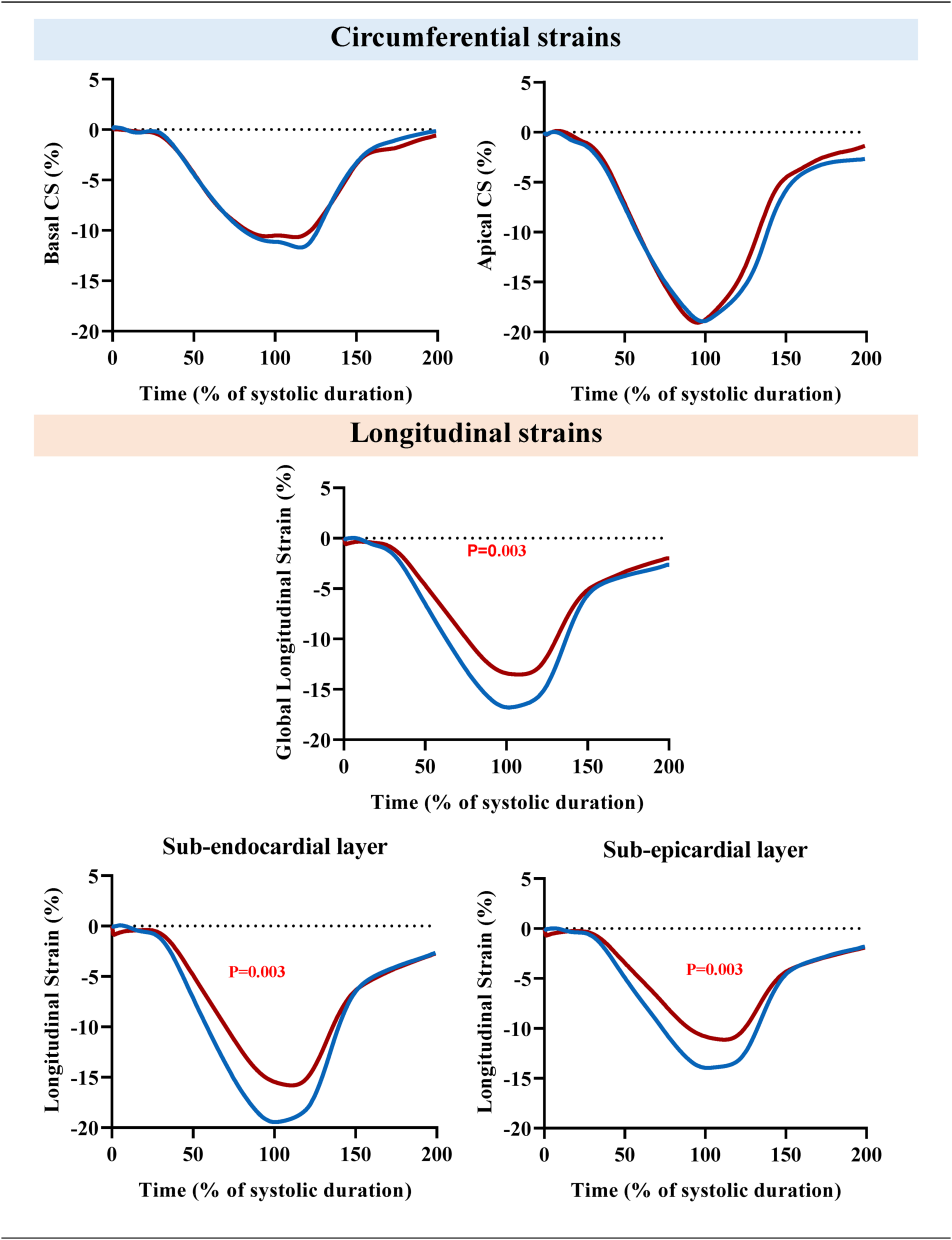
Longitudinal and circumferential strain. Longitudinal strain and circumferential strains (CS) in childhood lymphoma survivors (red curve) and controls (blue curve). Circumferential strain is displaced as basal and apical segments. Longitudinal strains are displaced as global longitudinal strain (GLS), sub‐endocardial and sub‐epicardial layers. Difference between groups: *p* < 0.05.

**FIGURE 3:**
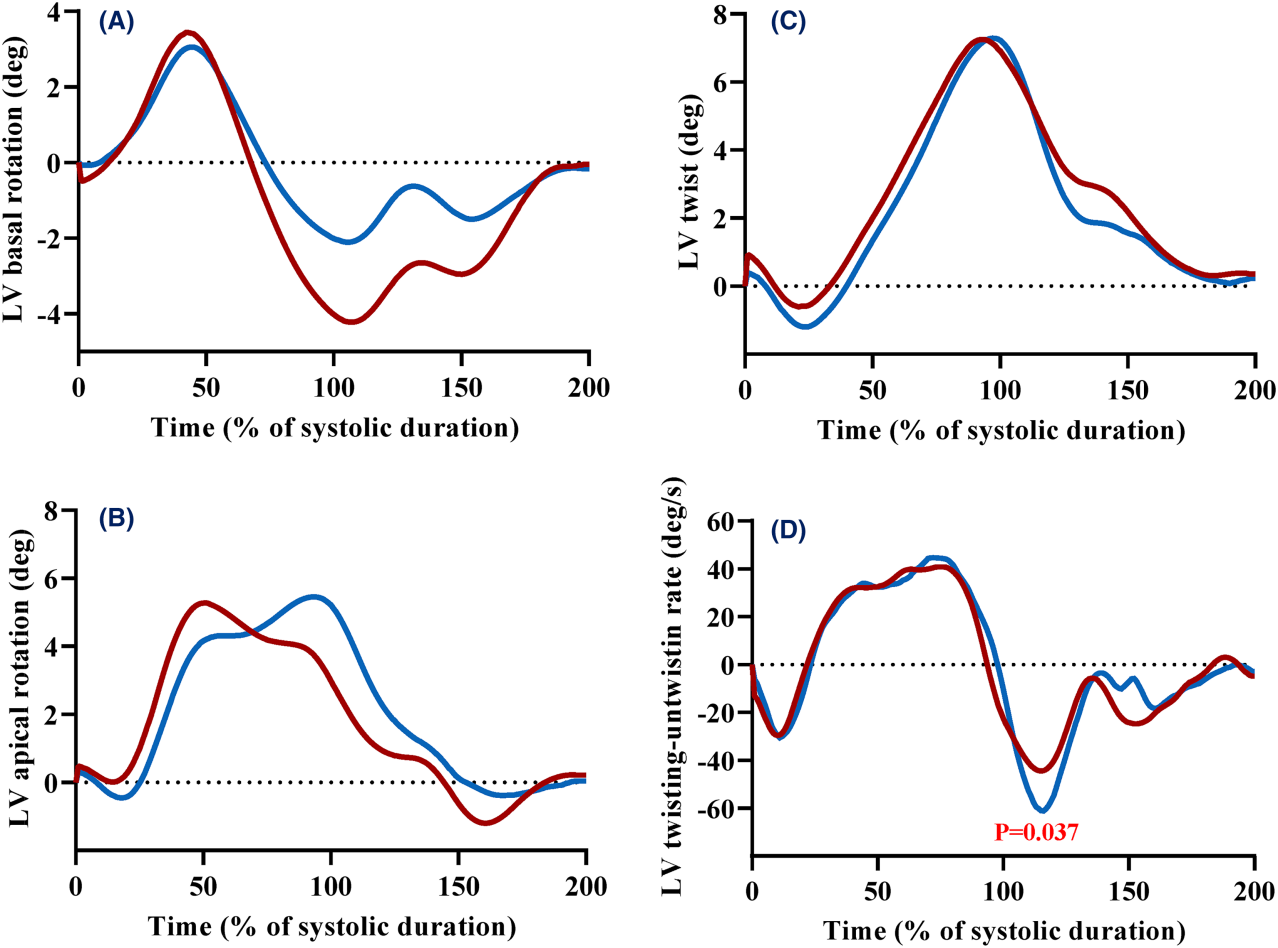
Left ventricular twisting mechanics. Twist and untwist mechanisms in childhood lymphoma survivors (red curves) and controls (blue curves). (A) Basal rotation, (B) apical rotation and (C) twist, function of time (% of systolic duration). (D) Twist –untwist rate, function of time (% of systolic duration). Difference between groups: *p* < 0.05.

LV strains are presented in Table 3 and Figure 2. GLS was diminished in CLS patients compared to controls, whereas no difference was observed on circumferential strains. We note a significant and similar difference in both sub‐endocardium and sub‐epicardium in CLS compared to controls. In CLS, the global strain rate values were significantly inferior and we note a significant difference in Diastolic GLS‐rate. LV twisting mechanics are presented in Table 3 and Figure 3. LV rotations and twist were similar between groups despite their patterns during the cardiac cycle appears slightly different. Peak untwisting rate, which occurred in very early diastole, was significantly lower in CLS compared to controls.

**TABLE 3:** Left ventricular strains.

	Patients (*n* = 16)	Controls (*n* = 15)
LV longitudinal strains		
GLS (%)		
Myocardium	−14.2 ± 2.5 ***	−17.1 ± 1.6
Sub‐endocardium layer	−16.6 ± 2.7 ***	−19.8 ± 2.4
Sub‐epicardium layer	−11.5 ± 2.6 **	−14.1 ± 1.9
Systolic GLS‐rate (.s^−1^)	−0.75 ± 0.11 **	−0.86 ± 0.06
Diastolic GLS‐rate (.s^−1^)	1.26 ± 0.33 **	1.54 ± 0.24
LV circumferential strains		
CS (%)		
Basal level	−11.3 ± 1.4	−11.8 ± 3.8
Apical level	−19.4 ± 6.4	−19.3 ± 5.6
Systolic CS‐rate (.s^−1^)		
Basal level	−0.79 ± 0.11	−0.82 ± 0.23
Apical level	−1.22 ± 0.45	−1.23 ± 0.29
Diastolic CS‐rate (.s^−1^)		
Basal level	1.15 ± 0.31	1.32 ± 0.42
Apical level	1.62 ± 0.39	1.82 ± 0.63
LV Twisting mechanics		
Rotations (deg)		
Basal level	−4.5 ± 2.6	−3.6 ± 1.9
Apical level	6.1 ± 2.6	6.6 ± 2.3
Twist (deg)	7.7 ± 3.2	8.1 ± 3.7
Untwisting rate (°/s^−1^)	‐ 61.2 ± 23.3 *	−82.5 ± 30.4

*Note*: Values are mean ± standard deviation. *: significantly different from Controls (*: *p* < 0.05; **: *p* < 0.01; *** *p* < 0.001).

Abbreviations: CS, circumferential strain; GLS, global longitudinal strain.

**FIGURE 4:**
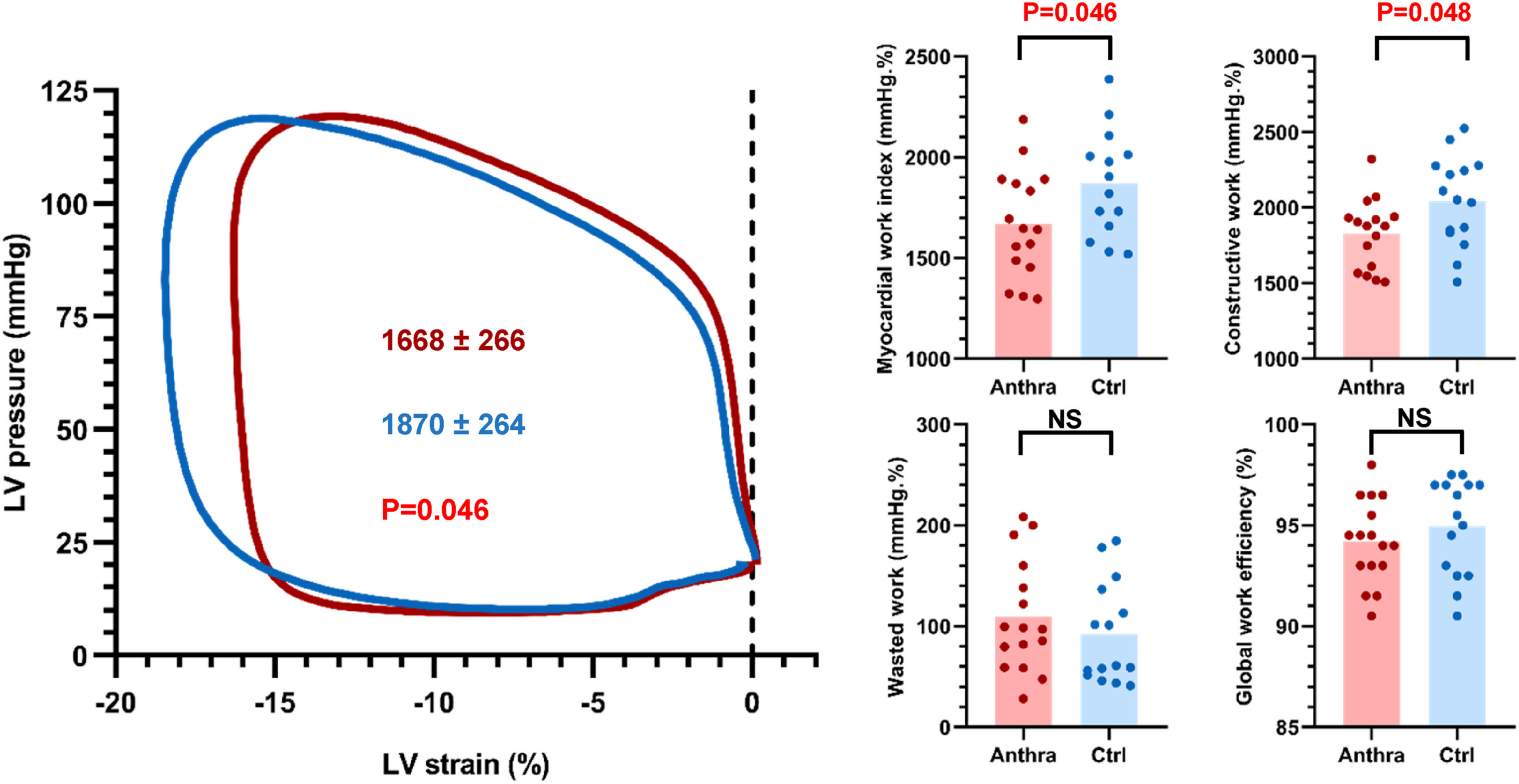
Myocardial work and its components. Noninvasive myocardial work estimation obtained from echocardiography. The left ventricle (LV) pressure–strain loop (PSL) is generated as a function of time of global longitudinal strain and systolic blood pressure, integrated to with the timing of aortic and mitral valve opening and closure. The area of the pressure–strain loop then provides the global myocardial work index (MWI) (at the left side). Global myocardial work efficiency (MWE) is then obtained by dividing the global constructive myocardial work (MCW) by the sum of both MCW and global myocardial wasted work (MWW). All global values are derived from averages of their segmental values.

**FIGURE 5:**
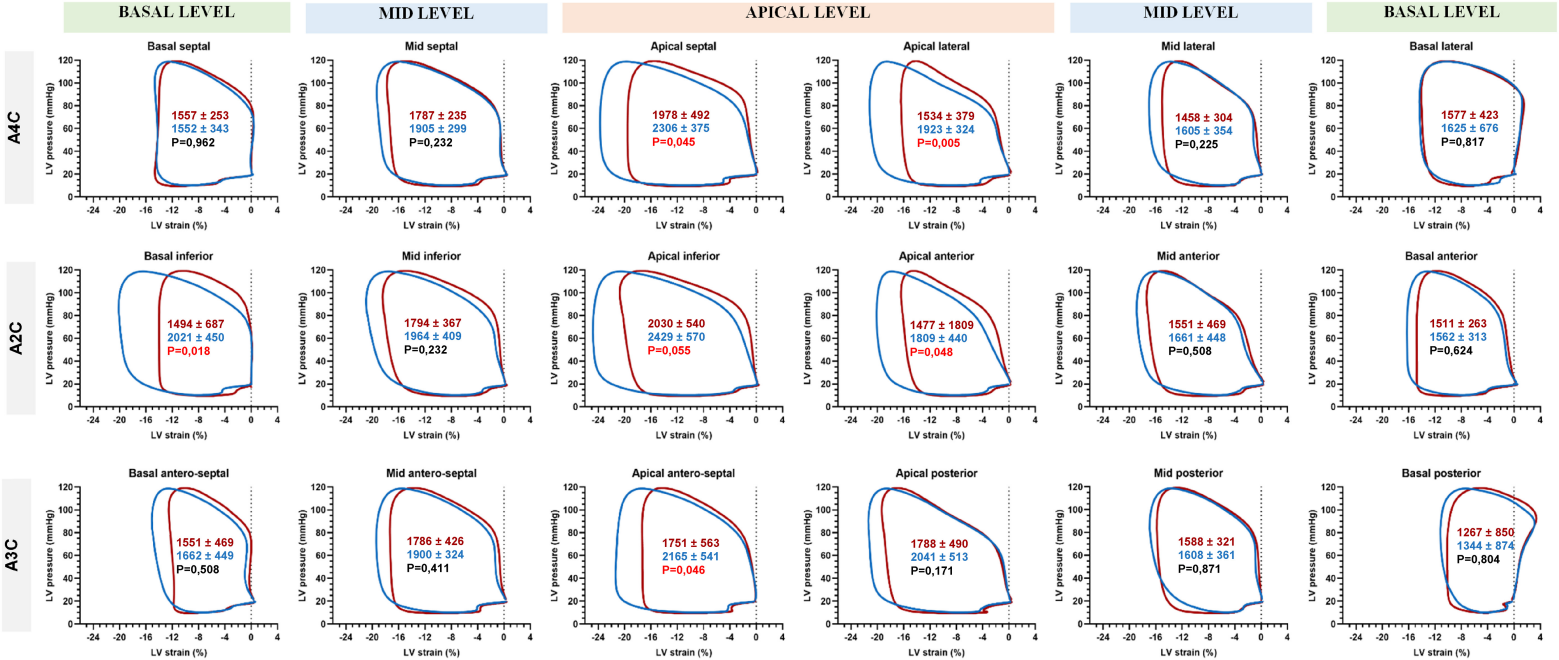
Segmental myocardial work index from the construction of left ventricular Pressure‐Strain loops. These 18 figures represent, for each line, the 3 (A3C), 2 (A2C), and 4 (A4C) chambers views, using the same coding with childhood lymphoma survivors (red curves) and controls (blue curves) on pressure strain loops (PSL). The area of the pressure–strain loops corresponds to the segmental myocardial work index (MWI). In each PSL segment, *p* < 0.05 indicates a significant difference with the corresponding segment of the control group.

MW data are presented in Table 4 and Figure 4. In CLS patients, the LV pressure‐strain loops (PSL) were shifted to the right, consequently to a decrease in their GLS. The MWI, calculated as the area of the PSL, was significantly different between groups (1668 ± 266 vs 1870 ± 264%. mmHg in CLS patients and controls, respectively). In CLS, segmental MWI values presented in Figure 5 were significantly lower in the apical segments, whereas no significant difference was shown in the mid and basal segments.

**TABLE 4:** Myocardial work and Left ventricular mechanical dispersion.

	Patients (*n* = 16)	Controls (*n* = 15)
Myocardial work		
Constructive work (%.mmHg)	1834 ± 262	2042 ± 298 *
Wasted work (%.mmHg)	123 ± 72	91 ± 50
Global work efficiency (%)	0.93 ± 0.03	0.95 ± 0.02
LV mechanical dispersion		
Delta 1–18	237 ± 149	145 ± 50 *
SD _18S_ (ms)	88 ± 33	58 ± 26 *

*Note*: Values are mean ± standard deviation. *: significantly different from Controls (*: *p* < 0.05).

## 4 DISCUSSION

Our study aimed to compare the cardiac function of CLS with age‐matched control subjects to determine the late anthracyclines cardiotoxicity using STE. In CLS, we observed: (1) a reduction of longitudinal strains in a similar manner in the sub‐endocardium and sub‐epicardium, without alteration of circumferential strains and twist, (2) a decrease of MW, mainly observed on apical segments, (3) an increase of LV intraventricular mechanical dispersion, but without significant alteration of MWW and global MWE.

### 4.1 Lower longitudinal strain without alteration of circumferential strains and twist in CLS

Our study confirmed that GLS, enabling a more sensitive analysis of systolic function than LVEF, was significantly lower in CLS than in controls although they received relatively low doses of anthracyclines. Of note, whereas GLS was decreased, LV CS, rotations and twist were unchanged in CLS. The LV twist plays an important role in promoting LV ejection during systole, therefore the maintenance of LV twist in CLS probably contributed to maintain their LVEF despite the decrease of the GLS. A similar pattern of impaired GLS without changes in CS was documented in young patients after chemotherapy,^13^, ^16^, ^18^, ^35^ but also in patients suffering of hypertrophic cardiomyopathy.^36^ Since the sub‐endocardial layer is principally composed of fibers oriented longitudinally, it has been often hypothesized that sub‐endocardial layer was more sensitive to myocardial diseases such as fibrosis. For example, lower CS is found in transmural myocardial infarction, differentiating it from subendocardial infarction.^13^ In adults treated with anthracyclines, previous studies observed that the late gadolinium cardiac magnetic resonance was enhanced specifically in the sub‐endocardium, suggesting the presence of fibrosis.^28^, ^37^ However, our results indicated that the decrease of LS was similar on both layers, that spoke in favor of an alteration of different zones of the myocardium wall rather than a specific alteration of the sub‐endocardial layer.

During systole, LV twist is induced by the contraction of the cardiomyocytes generating opposite rotations of the LV base and apex. This mechanism predominantly deforms the subendocardial fiber matrix, resulting in the storage of potential energy. Following untwist is associated with the release of these forces, which contributes to LV diastolic relaxation and early filling which onset and peak could be significantly delayed in diastolic heart failure.^38^ In our study, we observed that the untwisting rate was lowered in CLS despite their normal LV twist. This result has been described by others^17^, ^39^ and supports that underlying mechanisms of diastolic function were altered in CLS patients with a potential decrease of LV intrinsic myocardial relaxation.

### 4.2 Lower MW in CLS, especially on apical segments

Noninvasive PSL have been previously validated as a surrogate of MW and overcome the load‐dependency of EF and LS. Indeed, MW takes into account deformation as well as afterload through interpretation of strain in relation to noninvasive LV pressure (estimated by measurement of cuff pressures).^19^, ^40^, ^41^, ^42^ Assessments of MW were already done in preclinical and adult cohorts of patients.^21^, ^22^, ^43^ For example, MW has been assessed in ischemic cardiac disease, allowing the detection of subclinical coronary artery disease without any regional wall motion abnormalities, and has more sensitivity for myocardial oxygen consumption.^26^ In pediatric population, Zhan et al. recently demonstrated that global MWI enhanced hypertensive heart disease evaluation and cardiac dysfunction during and after chemotherapy follow‐up.^42^, ^44^ In our study, while systolic BP were similar between groups, the MWI was reduced in CLS, linked in a large part to their lower GLS.

The strength of our study was to assess not only global MW, but also segmental MW. Based on regional assessments on an 18‐segments model of the LV, we observed that MW was specifically decreased at the apical region. To our knowledge, no previous studies assessed the segmental MWI in CLS. This result was in agreement with previous study, showing impaired LS specifically in apical segments, in a cohort of childhood‐cancer survivors.^16^, ^45^, ^46^, ^47^ We also noticed a specific and significant decrease of MW on the basal inferior in CLS compared to controls, whereas other basal segments remained strictly unchanged. The clinical significance and the underlying mechanisms of this regional alteration remain unclear. Specific segmental modifications of MW have already been showed in adult populations for precising diagnosis, as a predictor of mortality by evaluation of its apex‐to‐base ratio in cardiac amyloidosis, or as short‐term prognosis markers in ischemic diseases or post‐therapeutic prediction response in cardiac resynchronization therapy.^40^ In our population and based on preclinical in‐vivo juvenile rat's study, our result could suggest starting fibrosis pattern in the apical regions in CLS.^48^ Apical deterioration might be also explained by the extent of sensitivity toward the LV apical vortex, which is formed by the helical fibers that exist without any surrounding circumferential muscle.^49^ Further investigations are needed to explore the role of the apex in myocardial mechanics and its specific implication in long‐term cardiac dysfunction in childhood‐cancer survivors. Our study underscored the ability of recent echocardiographic advancements, particularly those relying on regional left ventricular strains and MW, to detect subtle cardiac toxicity induced by anthracyclines in young patients with CLS. Despite the limited sample size in our study, these findings suggest that these novel assessments are particularly valuable and should be addressed in the follow‐up of such patients. This is supported by other recent studies that have highlighted MW as a highly sensitive evaluation of myocardial function during chemotherapy, aiding in the diagnosis of subclinical cardiotoxicity.^43^ The assessment of GLS has become widely employed across various pathological conditions. The incorporation of MW assessment necessitates the inclusion of only arterial pressure, simplifying its integration into routine clinical practice.

### 4.3 Higher intraventricular mechanical dispersion in CLS

Interstitial fibrosis was detected in microscopic slides of heart tissue from patients who had cardiac transplants after AIC, without significant cardiac hypertrophy.^50^ Myocardial fibrosis having adverse effects on excitability, sarcomeric organization, intracellular calcium homeostasis and myofilament Ca2+ sensitivity,^48^, ^51^ we hypothesized that these alterations could increase mechanical myocardial dispersion and decrease efficiency. Based on segmental STE analysis, we observed that SD18S and delta 1–18, both overall indexes of LV mechanical dispersion, were significantly increased in CLS compared to controls. Other studies also showed increased systolic intraventricular dyssynchrony index in childhood‐cancer survivors, with significant negative correlations with LVEF.^52^, ^53^ To complete the study of mechanical dispersion, we measured MCW and MWW to calculate MWE. In patients with increased mechanical dispersion, MWW is generally increased and consequently MWE is reduced. However, we did not find significant alteration of both MWW and MWE in CLS compared to controls. These findings were nevertheless concordant with Zhan et al. showing preserved MWE^42^ but the discrepancies in our results between increased mechanical dispersion and unchanged MWE remained not fully understood.

### 4.4 Study limitations

Our study has several limitations. First, this is a single‐center study including a small group of patients. However, despite the small sample size, our data clearly showed a significant impact of anthracycline on LV regional myocardial function assessed by 2D‐strain echocardiography and MW. Analyzing differences in myocardial function among subtypes of NHL in young adults post‐lymphoma with AIC would be of interest. However, due to the limited size of the study population, it was not feasible to incorporate the NHL subtype in the statistical analysis. Moreover, we assessed only the late effects of anthracycline, known to develop even years after completion of treatment, and it would be interesting to assess their cardiac function before and during the treatment. Finally, it would be helpful to link the increase of the LV mechanical dispersion observed in CLS with a potential increase of myocardial fibrosis from cardiac magnetic resonance imaging with late gadolinium enhancement.

## 5 CONCLUSION

With more CLS now reaching adulthood, understanding the adverse effects of chemotherapy and particularly anthracyclines on the cardiovascular system are of major interest. Our results highlighted that the late cardiac effects of chemotherapy based on low to moderate doses of anthracyclines were characterized by a decrease of MW, a new echocardiographic tool that is useful to detect early and subtle dysfunction in various pathologies. Based on a regional analysis, our data strongly supported that MW was diminished specifically in apical segments. Moreover, our regional analysis enabled an assessment of the intraventricular mechanical dispersion, which was higher in CLS compared to controls. All‐in‐one, our study strongly supported a late effect of chemotherapy based on anthracyclines on regional myocardial function and LV mechanical dispersion in adolescents and young adults. However, further studies would be helpful to evaluate the clinical significance of these cardiac remodeling and their potential long‐term adverse cardiac events, to propose an adapted follow‐up to CLS and evaluate the benefit of cardio‐protective therapies.

## AUTHOR CONTRIBUTIONS

**Mohamed Jaber:** Conceptualization (equal); data curation (equal); formal analysis (equal); investigation (lead); methodology (equal); writing – original draft (lead); writing – review and editing (equal). **Alexandre Armand:** Data curation (equal); methodology (equal). **Emmanuelle Rochette:** Methodology (equal); validation (equal). **Severine Monzy:** Investigation (equal). **Victoria Greze:** Project administration (supporting); writing – review and editing (supporting). **Justyna Kanold:** Supervision (supporting); writing – review and editing (supporting). **Etienne Merlin:** Supervision (supporting); writing – review and editing (supporting). **Justine Paysal:** Formal analysis (equal); investigation (equal); methodology (equal); supervision (lead); writing – review and editing (lead). **Stéphane Nottin:** Formal analysis (equal); investigation (equal); methodology (equal); supervision (lead); writing – review and editing (lead).

## FUNDING INFORMATION

The authors have no relevant financial or non‐financial interests to disclose.

## CONFLICT OF INTEREST STATEMENT

The authors declare no competing interests.

## ETHICS STATEMENT

Was granted by the Comité de Protection des Personnes Sud‐Est V (no. 21.02921.000036 cat 1).

## Data Availability

The datasets generated during and/or analysed during the current study are available from the corresponding author on reasonable request.
